# Local and landscape environmental heterogeneity drive ant community structure in temperate seminatural upland grasslands

**DOI:** 10.1002/ece3.9889

**Published:** 2023-03-19

**Authors:** Antonio J. Pérez‐Sánchez, Anett Schibalski, Boris Schröder, Sebastian Klimek, Jens Dauber

**Affiliations:** ^1^ Thünen Institute of Biodiversity Braunschweig Germany; ^2^ Biodiversity of Agricultural Landscapes Institute of Geoecology, Technische Universität Braunschweig Braunschweig Germany; ^3^ Landscape Ecology and Environmental Systems Analysis Institute of Geoecology, Technische Universität Braunschweig Braunschweig Germany; ^4^ Berlin‐Brandenburg Institute of Advance Biodiversity Research (BBIB) Berlin Germany

**Keywords:** environmental filtering, evenness, Formicidae, fourth‐corner models, meadows, pastures, response traits, species composition

## Abstract

Environmental heterogeneity is an important driver of ecological communities. Here, we assessed the effects of local and landscape spatial environmental heterogeneity on ant community structure in temperate seminatural upland grasslands of Central Germany. We surveyed 33 grassland sites representing a gradient in elevation and landscape composition. Local environmental heterogeneity was measured in terms of variability of temperature and moisture within and between grasslands sites. Grassland management type (pasture vs. meadows) was additionally included as a local environmental heterogeneity measure. The complexity of habitat types in the surroundings of grassland sites was used as a measure of landscape environmental heterogeneity. As descriptors of ant community structure, we considered species composition in terms of nest density, community evenness, and functional response traits. We found that extensively grazed pastures and within‐site heterogeneity in soil moisture at local scale, and a high diversity of land cover types at the landscape scale affected ant species composition by promoting higher nest densities of some species. Ant community evenness was high in wetter grasslands with low within‐site variability in soil moisture and surrounded by a less diverse landscape. Fourth‐corner models revealed that ant community structure response to environmental heterogeneity was mediated mainly by worker size, colony size, and life history traits related with colony reproduction and foundation. We discuss how within‐site local variability in soil moisture and low‐intensity grazing promote ant species densities and highlight the role of habitat temperature and humidity affecting community evenness. We hypothesize that a higher diversity of land cover types in a forest‐dominated landscape buffers less favorable environmental conditions for ant species establishment and dispersal between grasslands. We conclude that spatial environmental heterogeneity at local and landscape scale plays an important role as deterministic force in filtering ant species and, along with neutral processes (e.g., stochastic colonization), in shaping ant community structure in temperate seminatural upland grasslands.

## Graphical abstract

We followed a multiscale approach to assess the effect spatial environmental heterogeneity on ant community structure seminatural grasslands. Our results showed that soil moisture heterogeneity, extensively grazing management, topographic exposition, and diversity of land cover types shape ant community composition and evenness. Community structure response to environmental heterogeneity was mediated by a small set of morphological, ecological and life history species traits. This study showed that spatial environmental heterogeneity at local and landscape scale plays an important role driving ant community structure in temperate seminatural upland grasslands.

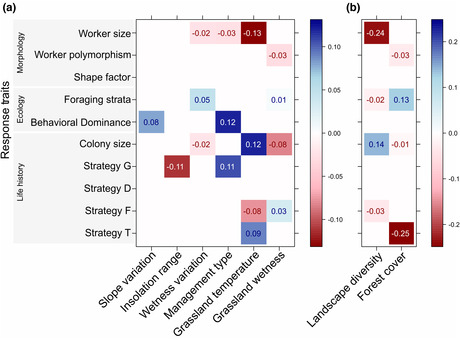

## 1 INTRODUCTION

Identifying the mechanisms underlying community assembly remains a central challenge in ecology. Ecological theory assumes that the dynamics and composition of communities are driven by the combined effects of environmental filtering (abiotic conditions), biotic conditions (inter‐ and intraspecies interactions), neutral processes (dispersal limitations), and historical contingencies (speciation) at multiple spatial scales (Cavender‐Bares et al., 2009; Götzenberger et al., 2012; Ovaskainen et al., 2017). Classical niche differentiation assumes environmental filtering and biotic interactions (mainly competition) as major mechanisms structuring local communities, selecting species with specific environmental requirements that allow them to survive and persist at a given location, but culling species unable to tolerate such conditions (Cadotte & Tucker, 2017; Kraft et al., 2015). However, the “filtering” effect of the abiotic environment is sensitive to the spatial scale and therefore intimately related to the spatial heterogeneity of the environment (Kraft et al., 2015). Defining the spatial extent (local, landscape, and regional) is therefore key to properly address the role of the environment and its variability in the filtering process of ecological communities (Cadotte & Tucker, 2017; Kraft et al., 2015).

Environmental heterogeneity is a ubiquitous driver of ecological processes in natural and seminatural systems (Costanza et al., 2011; De Bello et al., 2013; Stein et al., 2014). In a broad sense, environmental heterogeneity refers to all aspects of spatial heterogeneity, complexity, diversity, structure, or variability in abiotic and biotic environmental conditions (Stein et al., 2014; Stein & Kreft, 2015) and is regarded as a primary mechanism explaining diversity patterns and species coexistence (Costanza et al., 2011; Melbourne et al., 2007; Tilman, 1982). Spatial environmental heterogeneity is thought to promote species diversity through three major mechanisms (Stein et al., 2014; Stein & Kreft, 2015): (i) an increase of gradients or variability in the environment (with regard to the amount of resources, habitat types or structural complexity) should increase available niche space and allow more species to coexist; (ii) more heterogeneous habitats are more likely to provide refuge from adverse environmental conditions, promoting species persistence; and (iii) the probability of speciation events resulting from isolation or adaptation to diverse environmental conditions should increase with environmental heterogeneity. There is substantial evidence demonstrating that heterogeneity in abiotic and biotic environmental conditions plays a significant role in structuring communities by either deterministic and/or stochastic processes at local and landscape scale (Bar‐Massada et al., 2014; Brown et al., 2013; Götzenberger et al., 2012). Although there is widespread empirical evidence supporting a positive effect of environmental heterogeneity on species diversity (Stein & Kreft, 2015) and functional diversity (Price et al., 2017; Stark et al., 2017), the extent and generality of this positive relationships have been questioned by several studies (e.g., Gazol et al., 2013; Laanisto et al., 2013; Tamme et al., 2010).

The role of environmental conditions and heterogeneity in structuring ant communities (taxonomically and functionally) has been frequently addressed by means of climatic and habitat factors (Bernadou et al., 2015; Sanders et al., 2007; Sarty et al., 2006), particularly at regional and global scales (Arnan et al., 2017; Gibb & Parr, 2010; Lassau et al., 2005). For example, environmental filtering has been suggested as the main ecological mechanism structuring European ant communities at continental and biogeographic scale (Arnan et al., 2017; Boet et al., 2020), while habitat complexity and abiotic variation along environmental gradients have been shown to shape taxonomic and functional diversity of ants in warm‐temperate Mediterranean regions (Arnan et al., 2014; Blatrix et al., 2016). A recent study conducted in differently structured urban green spaces further indicated that structural complexity of the local vegetation can act as an environmental filter, driving ant communities in terms of species numbers and functional traits (Nooten et al., 2019). Comparatively few studies have been conducted in managed temperate grasslands by directly addressing the environment‐community structure relationship at different spatial scales (Dahms et al., 2010; Dauber et al., 2003; Dauber & Wolters, 2005), and even fewer have included a functional trait‐based approach (Heuss et al., 2019; Scharnhorst et al., 2021; van Noordwijk et al., 2012).

Ants are an important and omnipresent component of biodiversity in grasslands and constitute major aboveground generalist predators (Sanders & van Veen, 2011; Seifert, 2018; Wills & Landis, 2018). They are considered ecosystem engineers, directly or indirectly controlling many ecosystem processes by altering physical, chemical, and biological soil properties at their nesting sites (Frouz & Jilková, 2008; Sanders & van Veen, 2011; Wills & Landis, 2018). In European temperate grasslands, ant communities have mostly been described and analyzed with regard to land‐use impact on species richness and abundance (e.g., Dahms et al., 2005; Pérez‐Sánchez et al., 2018; Pihlgren et al., 2010). Although there is solid evidence suggesting that land‐use intensification (increased grazing, mowing, and fertilization) decreases ant richness in temperate grasslands (Heuss et al., 2019), many studies suggest that local differences in microclimate and soil conditions influence ant communities more strongly than direct management practices (Dahms et al., 2005; Pérez‐Sánchez et al., 2018; Seifert, 2017). In fact, a site‐dependent response of ant communities to management is a strikingly common outcome in almost all research efforts so far, even in large‐scale studies across wider geographic gradients (Heuss et al., 2019; Pérez‐Sánchez et al., 2018; Seifert, 2017). This site‐dependent pattern has been explained by how management practices or their absence affect biotic (e.g., physical structure of vegetation) and abiotic (e.g., microclimate heterogeneity) conditions for ants in a local context (Pérez‐Sánchez et al., 2018; Seifert, 2017), which can also be understood as how environmental heterogeneity drives ant community composition at within‐site or local scale. Consequently, environmental variability, including heterogeneity induced by management, at local (within grasslands) and landscape (surroundings) scale may play a major role in shaping ant communities in European temperate grasslands.

Here, we assessed the effects of local and landscape spatial environmental heterogeneity on ant community structure in temperate seminatural upland grasslands of Central Germany. We used “environmental heterogeneity” as an umbrella term (sensu Stein & Kreft, 2015) to describe the variability in temperature, soil moisture, and management type (pasture vs. meadow) within and between grassland sites (local environmental heterogeneity), as well as the complexity of habitat types in the surrounding landscape (landscape environmental heterogeneity). As descriptors of ant community structure, we focused on species composition in terms of nest density, community evenness, and selected ant species traits following a fourth‐corner model approach (Brown et al., 2014). We addressed the following questions:

1. Does ant species composition respond to local (within and between grassland sites) and landscape (grassland site surroundings) environmental heterogeneity?
2. Is ant community evenness (independently of species identity) positively affected by environmental heterogeneity at both spatial scales?
3. Which species traits mediate the response of ant community structure to environmental heterogeneity?

We expect ant community structure to be determined by environmental heterogeneity at both local and landscape scale, with a differential response on species composition but an increase in community evenness along with environmental heterogeneity. Similarly, we expect that the response of ant community to environmental heterogeneity can be explained by ecological mechanisms involving not only a set of species morphological traits but also species ecological and life history attributes (Gibb et al., 2015; Retana et al., 2015; van Noordwijk et al., 2012).

## 2 MATERIALS AND METHODS

### 2.1 Study area

**FIGURE 1:**
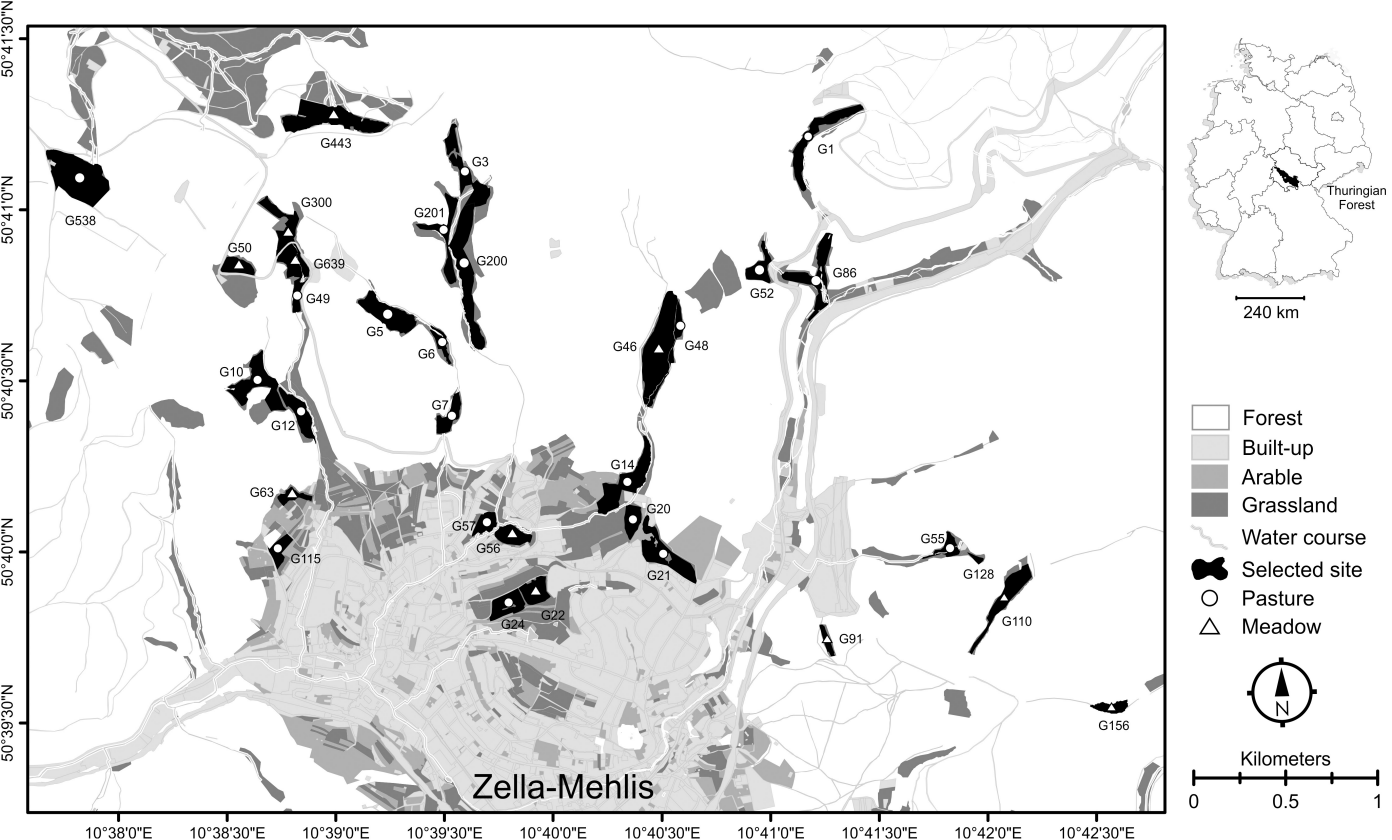
Study area in the Thuringian Forest, Central Germany. The map shows the distribution of the 33 selected sites (black) within the grassland complexes (dark gray) and forest matrix (white). Codes within map indicate the respective grassland site ID.

The study area is situated in the Thuringian Forest National Park, in the vicinity of the city of Zella‐Mehlis, Germany (Figure 1). The area is characterized by a steep elevation gradient ranging from 450 m (Zella‐Mehlis) to c. 900 m above sea level (highest mountain peak). Mean annual temperature is 5°C, and mean annual precipitation is 1100 mm (Deutscher Wetterdienst, 2017). The landscape is dominated by spruce forest (65%), followed by built‐up areas (15%), extensively managed grasslands (11%), and small fragments of arable fields (3%). Grasslands are predominantly located in the surrounding of the city or along mountain valleys on steep slopes. Some grassland sites are isolated from each other by forests and have been traditionally used for haymaking, while others are connected by rotational extensive grazing to allow moving livestock from one pasture to the next. We selected 33 grassland sites that represent a gradient in elevation and landscape composition in the region (Figure 1), with mean topographical distance of 2100 m between grassland sites (edge to edge; min = 30 m, max = 6900 m). All selected sites were managed either as extensive pastures or extensive meadows, with no history of management intensification (in terms of increased livestock density or mowing rate, mineral fertilizer, or pesticide use) or land abandonment (in terms of woody plant encroachment) in the last decades.

### 2.2 Ant survey and nest density

Ant assessment was based on Seifert's (2017) sampling method for non‐arboreal ant species in Central Europe. This method is focused on direct localization of nest sites and determination of nest density per unit area, where species number found in a certain habitat is a function of sampling effort quantified by square meters of searched ground area (Seifert, 2017). Seifert (2017) approach for ant survey is particularly suitable in European grasslands where most of species have visible nest entrances aboveground and has an edge over other traditional collecting methods as it overcomes potential sampling‐bias caused by differences on workers foraging distances and species colony size. We choose this ant survey method as it provides a standardized whole‐community characterization at grassland scale that fits consistently with our environmental heterogeneity assessment.

Ant survey procedure consists of direct localization of workers and nests within a spatially nested scheme covering three levels of search effort in a specific area: an intensive scrutiny (*S*‐) search performed at each square decimeter of soil and substrate (vegetation) surface within a smaller area (*S*‐ sampling area); a quick (*Q*‐) but sustained search on ground surface performed within a larger area (*Q*‐ sampling area); and a spot inspection (*SI*‐) in the most promising habitats for nests in the surroundings of the *Q*‐ areas (Seifert, 2017). The *S*‐ search aims to detect nests of small species with hidden nests or species with small territories, while the *Q*‐ search reflects realistic nest densities of larger species with lower nest densities but larger territories (Seifert, 2017). The SI sampling allows discovering nests of rare species such as social parasites of *Lasius* or *Formica* genera (Seifert, 2017). We employed fixed dimensions of 64 m^2^ for *S*‐ sampling areas and 400 m^2^ for *Q*‐ sampling areas, while *SI* areas covered *c*. 900 ± 82 m^2^. The combination of these three levels constituted a sample unit referred hereafter as Seifert‐plot. Time expenditure for ant searching in *S*‐ sampling areas varied from 30 to 60 min and up to 180 min for the entire Seifert‐plot. Time variation within *S*‐ sampling area depended on habitat structure as areas with complex structure and many microhabitats require more sampling time than open areas with less complex structure where nests entrances are more visible (Seifert, 2017). Since sampling effort in Seifert's (2017) method is based on square meters searched, variation on time investment within *S*‐ sampling area do not affect probability of finding ants as long as the scrutiny search is performed accordingly. Recording of foraging workers and nests was performed sequentially from *S*‐ to *Q*‐ and *SI*‐ areas, and up to 10 workers per nest were collected after finishing each sampling area. One to three Seifert‐plots were established per site depending on grassland size, presence of cattle, and limited access due to rugged topography or flooded areas (Table S1). All Seifert‐plots were searched between 08:00 and 18:00 h local time in August 2017. All specimens collected were fixed in ethanol 90% and determined to species level using identification keys in Seifert (2018).

Nest counts from each Seifert‐plot component were combined into a final integrated species‐specific density following Seifert's (2017) method, which represents the nest density of a species within 100 m^2^. This integrated density is determined by allocating a species into a given *recording group*, which is a generalization of how perceptible a nest is based on the ant species biology (Seifert, 2017). *Recording groups* describe the probability of finding a nest in each sampling level (*S*‐, *Q*‐, and *SI*‐; Seifert, 2017, 2018). Thus, a final integrated density value per grassland site was calculated as the sum of nests found in *S*‐, *Q*‐, and *SI*‐ search levels divided by a *pseudo‐area* of the recording group to which a particular species belongs (Seifert, 2017). *Pseudo‐areas* are a weighting parameter calculated for each recording group separately and provide a measure of the total intensity of investigation on a certain Seifert‐plot (Seifert, 2017). Detailed information regarding integrated density calculation and sampling completeness assessment is provided in Box S1 of the Appendix S1. Data from each Seifert‐plot were pooled at grassland site level, and a species by site matrix was constructed for further community analysis using integrated species‐specific density (hereafter referred simply as nest densities) as entries.

### 2.3 Environmental heterogeneity measures

We divided environmental data into local and landscape environmental heterogeneity measures. The former subset comprised variability measures related to abiotic and biotic environmental conditions within and between grassland sites, while the latter subset included biotic land cover heterogeneity measures in the surrounding landscape of each grassland site (Stein et al., 2014; methodological details are provided in Table 1).

**TABLE 1:** Local and landscape environmental heterogeneity measures (with minimum, median, and maximum values) used as predictors of ant community structure (*n* = 33).

Variable	Description	Unit	Remarks	Values		
				Min	Med	Max
Local environmental heterogeneity						
Within‐site heterogeneity measures						
Elevation range	Elevation range	m	Describes local climate gradient	7.3	29.7	97
Aspect range^a^	Surface aspect range	rad	Describes micro‐topographic structures exposure	1.2	2.5	6.3
Slope variation^b^	Coefficient of variation of slope	%	Describes micro‐topographic structures and variability	25.4	31.5	53
Insolation range^c^	Surface solar radiation range	kWh m^−2^ d^−1^	Proxy for soil temperature range	0.1	0.5	0.9
Wetness variation^d^	Coefficient of variation of topographic wetness index	%	Proxy for soil moisture variability	3.7	11.7	30.1
Management type^e^	Grassland management type	–	Describes vegetation structural heterogeneity	Pasture *n* = 21		Meadow *n* = 12
Between‐site heterogeneity measures						
Area^f^	Total area	ha	Account for unequal area (area‐Env. Het. relationship)	0.21	1.70	6.31
Grassland temperature^g^	Mean air temperature	°C	Habitat temperature filter	20.2	25.6	29.4
Maximum insolation^c^	Maximum solar radiation	kWh m^−2^ d^−1^	Proxy for maximum soil temperature	5.08	5.42	5.62
Grassland wetness	Mean topographic wetness index	–	Proxy for grassland soil moisture	7.1	9.03	16.5
Landscape environmental heterogeneity						
Forest cover	Percentage of land cover types in the grassland sites surroundings (geodesic buffers of 250 m from the edge)	%	Describes between‐habitat heterogeneity	0.56	77	96.1
Grassland cover				1.78	17.1	41.1
Arable cover				0.69	2.77	20.8
Built‐up cover				0.01	0.13	50
Landscape diversity	Shannon index of diversity for land cover types	–	Proxy for land cover heterogeneity	0.19	0.71	1.41

^a^ Surface aspect measured as the exposure of the slope within each grassland site.

^b^ Slope was calculated as the inclination degree of the grassland surface.

^c^ Solar energy input at grassland surface (summer time) calculated with the Area Solar Radiation tool in ArcGIS 10.5.1.

^d^ Tendency of water distribution on grassland surface calculated with the System for Automated Geoscientific Analyses (SAGA 2.3.2) module in QGIS version 2.18.16.

^e^ Pastures grazed by cattle at low stocking rate (0.6 livestock units per hectare) between May to August; meadows traditionally mown once a year (light machinery) between mid and end of August.

^f^ Grassland area was mapped and estimated using a Getac F110 G2 device (GPS: SiRFstarIV).

^g^ Grassland air temperature was derived using Tinytag Plus 2 (TPG‐4500) sensors located at 1.5 m above ground during mid‐July, July–August and late‐August 2017.

At the local scale, we calculated either the coefficient of variation [(standard deviation/mean)*100] or range (max–min) of elevation, surface aspect, surface slope, surface insolation and topographic wetness index within‐site using a grid‐based digital elevation model (10 m spatial resolution) derived from LiDAR data (German Office for Surveying and Geoinformation; Table 1). We additionally treated local grassland management type (pasture or meadow) as an environmental heterogeneity measure since both grazing and mowing lead to different levels of structural heterogeneity in vegetation and soil within grasslands (Tälle et al., 2016). In order to incorporate the role of mean climatic conditions as local environmental filters for ants, we further considered mean air temperature, maximum surface insolation, and mean topographic wetness index values per grassland site (Seifert, 2017). Since area and environmental heterogeneity are often closely related (Stein et al., 2014), we additionally included grassland site area as a local variable to account for any confounding effect between both parameters. The first six environmental measures involving coefficient of variation and range values (and categorical output) were highlighted as *within‐site heterogeneity measures* to showcase internal abiotic conditions variability per each site (environmental heterogeneity sensu Stark et al., 2017); while the last four variables involving mean, total or maximum values were highlighted as *between‐site heterogeneity measures* to showcase abiotic conditions variability among surveyed grassland sites (environmental means sensu Stark et al., 2017). All variables were pooled and treated as local environmental heterogeneity measures (Table 1).

To characterize environmental heterogeneity at the landscape scale, we calculated the landscape composition using digital thematic maps from German Real Estate Cadastre Information system (ALKIS) at a fine spatial resolution (1:5000). We calculated the percentage of land covered by forests, grasslands, arable land, and built‐up areas (roads, urban, and industrial areas) within a geodesic buffer of 250 m (edge to edge) for each grassland site (Table 1). Such buffer size provides an adequate spatial scale for evaluating the effect of landscape composition on ants in agricultural landscapes (Dauber et al., 2003). We additionally estimated the Shannon index of diversity based on land cover type areas as proxy of landscape heterogeneity (Table 1).

### 2.4 Ant traits

According to our research questions, we focused on species traits that may reflect ant responses to biotic or abiotic environmental conditions (Lavorel & Garnier, 2002; Violle et al., 2007). We selected seven response traits representing ant morphology, ecology, and life history in Central European grasslands (Table 2; methodological details are provided in Table S2). Response trait selection was based on previous findings by van Noordwijk et al. (2012), Retana et al. (2015), Seifert (2017), and Heuss et al. (2019). Trait data were pooled into a species by trait matrix for further statistical analysis (Brown et al., 2014).

**TABLE 2:** Ant response traits used in the fourth‐corner analysis. Species traits represent ant morphology, ecology and life history features in Central European grasslands (more detail in Table S2).

Traits	Response interpretation
Morphology	
Worker size	Both body size measures reflect physiological and ecological features of insect species and are strongly related to habitat disturbance and climate (Gibb et al., 2015; Retana et al., 2015; Seifert, 2017)
Worker shape factor	
Worker polymorphism	Caste specialization provides advantages in resource exploitation against environmental fluctuations (Arnan et al., 2017; Gibb et al., 2015; Retana et al., 2015)
Ecology	
Behavioral dominance	Indicates species ability to gain access to and exploit food resources, as well as defend nest and foraging territory (Savolainen et al., 1989; Savolainen & Vepsäläinen, 1988)
Foraging strata value	Indicates the vertical extent covered per species in grassland habitats (Heuss et al., 2019)
Life history	
Colony size	Size of colony is used as proxy of ecological success in social species (Bourke, 1999; Retana et al., 2015)
Life history strategy	Four types grouping species based on colony reproduction, development, dispersal and synchronization: G‐ generalist, D‐ poor dispersers, F‐ food limited, T‐ temperature limited (van Noordwijk et al., 2012)

### 2.5 Statistical analysis

All statistical analyses were performed using R version 3.6.3 (R Development Core Team, 2020). Prior to the analysis, local and landscape environmental heterogeneity measures and traits were checked for collinearity issues using the pairwise Pearson's correlation coefficient *r* (Dormann et al., 2013). All variables showed |*r*| < .7 and were therefore considered as predictors in further analyses (Figure S1).

#### 2.5.1 Community data exploration and evenness calculation

To illustrate variation in ant species composition within and between grassland sites, we performed a hierarchical cluster analysis using Euclidean distance and Ward's minimum variance as agglomeration method (Murtagh & Legendre, 2014). We used the average silhouette criterion for internal validation of the cluster analysis (Kaoungku et al., 2018; Rousseeuw, 1987). The cluster analysis was calculated and validated using vegdist and hclust functions of Vegan R package version 2.5‐0 (Oksanen et al., 2019), and fviz_silhouette and eclust functions of the factoextra package version 1.0.6 (Kassambara & Mundt, 2019).

Community evenness per grassland site was assessed by means of the relative evenness proposed by Jost (2010), which represents the amount of evenness relative to the minimum and maximum possible for a given richness. We used the relative logarithmic evenness (RLE) based on the diversity of order *q* also known as “true diversities” or “Hill numbers” (hereafter ^*q*^ *D*; Jost, 2006, 2010). The order (*q*) of a diversity (*D*) indicates the sensitivity of the measurement to common and rare species (Jost, 2006): for *q* = 0, the resulting value of a diversity (^0^ *D*) is indifferent to species frequencies, favoring rare species by giving the same weight to all species in the community (e.g., species richness); for *q* = 1, all species are weighted with their exact frequencies, and neither rare nor common species are favored in the resulting diversity value (^1^ *D*; e.g., exponential of Shannon entropy index); and for *q* = 2, species are weighted with the most frequent species in the community which favors more abundant species (^2^ *D*; e.g., inverse of Simpson index). We chose the RLE of orders 0 and 2 (RLE_0,2_ = ln ^2^ *D*/ln ^0^ *D*), as it represents the proportion of the most abundant species in a community (Jost, 2010). Both ^0^ *D* and ^2^ *D* are measures of effective number of species that satisfy the replication principle and account for the uniqueness of each species composing a community (Gotelli & Chao, 2013; Jost, 2006). RLE_0,2_ values range between 1 (perfectly even community) and nearly 1/^0^ *D* (community dominated by one species; Jost, 2010). Calculations of ^*q*^ *D* were based on species nest density (see section 2.2) using PAST software version 3.25 (Hammer et al., 2001).

#### 2.5.2 Testing community structure—Environmental heterogeneity relationship

To test whether local and landscape environmental heterogeneity measures affect ant community structure, we applied generalized linear models for multivariate data (Wang et al., 2012). This method fits individual GLMs for each species using a common set of explanatory variables and implements resampling‐based hypothesis testing to make community‐level and taxon‐specific inferences about which environmental factors are associated with such multivariate data (Wang et al., 2012; Warton et al., 2015). We fitted multivariate GLMs for both subsets of environmental heterogeneity measures (Table 1) as predictors and species‐site abundance (nest counts) as response variable, using a negative binomial distribution and a log‐link function. An offset term equals to mean *pseudo‐area* (log‐transformed) per grassland site was used in every model in order to interpret results in terms of nest density rather than raw nest counts (Warton et al., 2015). We used a backward stepwise model selection based on Akaike's Information Criteria (AIC) in order to find the most parsimonious model for statistical inference (Burnham & Anderson, 2002). The model with the smallest AIC was selected, and possible interactions were considered (Burnham & Anderson, 2002). Dunn–Smyth residuals plotted against fitted values were used to check model assumptions (Wang et al., 2012). We used an analysis of deviance based on likelihood ratio statistics (LR) to test the significance of each predictor variable on ant community (“sum‐of‐LR” statistic; Wang et al., 2012; Warton et al., 2015). To account for correlation in nest density across species, we used parametric bootstrapping (Monte Carlo, 999 bootstrap resamples), a method with good performance for small samples (*n* < 32; Warton et al., 2017). Multivariate GLMs and significance testing were implemented using the functions manyglm and anova.manyglm in R package mvabund version 4.0.1 (Wang et al., 2019).

To assess the effect of local and landscape environmental heterogeneity measures on ant community evenness, we implemented beta regression models (Ferrari & Cribari‐Neto, 2004). Beta regression is a well‐suited approach for modeling data that are bounded to the standard unit interval (0, 1) such as rates and proportions, and whose observations do not reach the limits of the interval (Ferrari & Cribari‐Neto, 2004). We fitted beta regression models for both predictor variable subsets using RLE_0,2_ as a response variable with a logit link function. Model selection and statistical inference were conducted as described above. Model assumptions were visually inspected in diagnostic plots of residuals and normal QQ‐plots (Zuur et al., 2010). Beta regression models were calculated using the betareg function in R package Betareg version 3.1‐2 (Cribari‐Neto & Zeileis, 2010), while the lrtest function from the R package lmtest was used for testing likelihood ratios on nested models (Zeileis & Hothorn, 2002).

#### 2.5.3 Fourth‐corner models

To quantify species responses to local and landscape heterogeneity measures and to understand their relationship with critical response traits, we applied a predictive fourth‐corner approach proposed by Brown et al. (2014). This approach fits a single model to predict abundances across several taxa as a function of environmental variables, taxa (species) traits, and their interaction (Brown et al., 2014; Löbel et al., 2018). Fourth‐corner models were fitted using count data (back‐transformed densities) and negative binomial distribution with a LASSO penalty estimated via cross‐validation. The LASSO penalty automatically sets to zero any term in the model that does not explain any variation in species response (Brown et al., 2014). We first fitted a single predictive model for all ant species at all grassland sites assuming different environmental responses for different species (not attempting to explain responses using traits). This can be understood as a multispecies distribution model (multivariate SDM; Brown et al., 2014; Wang et al., 2019). Second, we fitted a fourth corner model by adding the trait term to the model equation in order to evaluate how differences in the responses of species to local and landscape environmental heterogeneity measures were mediated by traits. In order to assess the interaction strength and importance of each predictor variable on species density and traits, we plotted the standardized coefficients from resulting models (Brown et al., 2014; Gibb et al., 2015). Multivariate SDMs and fourth‐corner models were fitted using the traitglm function from mvabund package version 4.0.1 (Wang et al., 2019).

## 3 RESULTS

A total of 16 species, six genera, and 465 nests were recorded in 32 grassland sites. At one grassland site (G20, Figure 1) neither ants nor nests were detected. The average number of detected species was 4.15 ± 0.72 (mean ± 95% CI) per grassland site (min = 1, max = 9; Figure S2.1). Total or whole‐community nest density varied between 0.22 and 22.7 with an average of 7.85 ± 2.29 nest/100 m^2^ per grassland site (Figure S2.1). Species with the highest nest density were *Myrmica scabrinodis*, *Myrmica rubra*, *Lasius niger*, *Formica fusca*, and *Lasius flavu*s (Figure S2.1). RLE_0,2_ values showed a gradient in ant community evenness across grassland sites from even (RLE_0,2_ ≥ 0.65, 54% of sites) to uneven communities (0.45 < RLE_0,2_ < 0.65, 35% of sites), with few sites having dominated communities (RLE_0,2_ ≤ 0.45, 11%; Figure S2.1).

Cluster analysis revealed a pattern of species composition within grassland sites related to management type, whole‐community nest density, different levels of evenness, and identity of the most dense species or group of species (for details, see Box S2). First cluster division generated one group of communities with high nest density in sites predominately managed as pasture and another group of communities with low‐density located in sites either managed as pasture or meadow (Figure S2.1). Further divisions generated grassland sites clusters with: uneven communities with high density of *M. scabrinodis* (cluster 1), even communities of rather low nest density (cluster 2), communities with intermediate densities of *M. scabrinodis* (cluster 3), communities with high nest densities of *L. flavus* (cluster 4), communities where *M. rubra* was the most dense species (cluster 5), and communities with intermediate densities of *M. scabrinodis* but relatively high densities of *L. niger* (cluster 6; Box S2).

**FIGURE 2:**
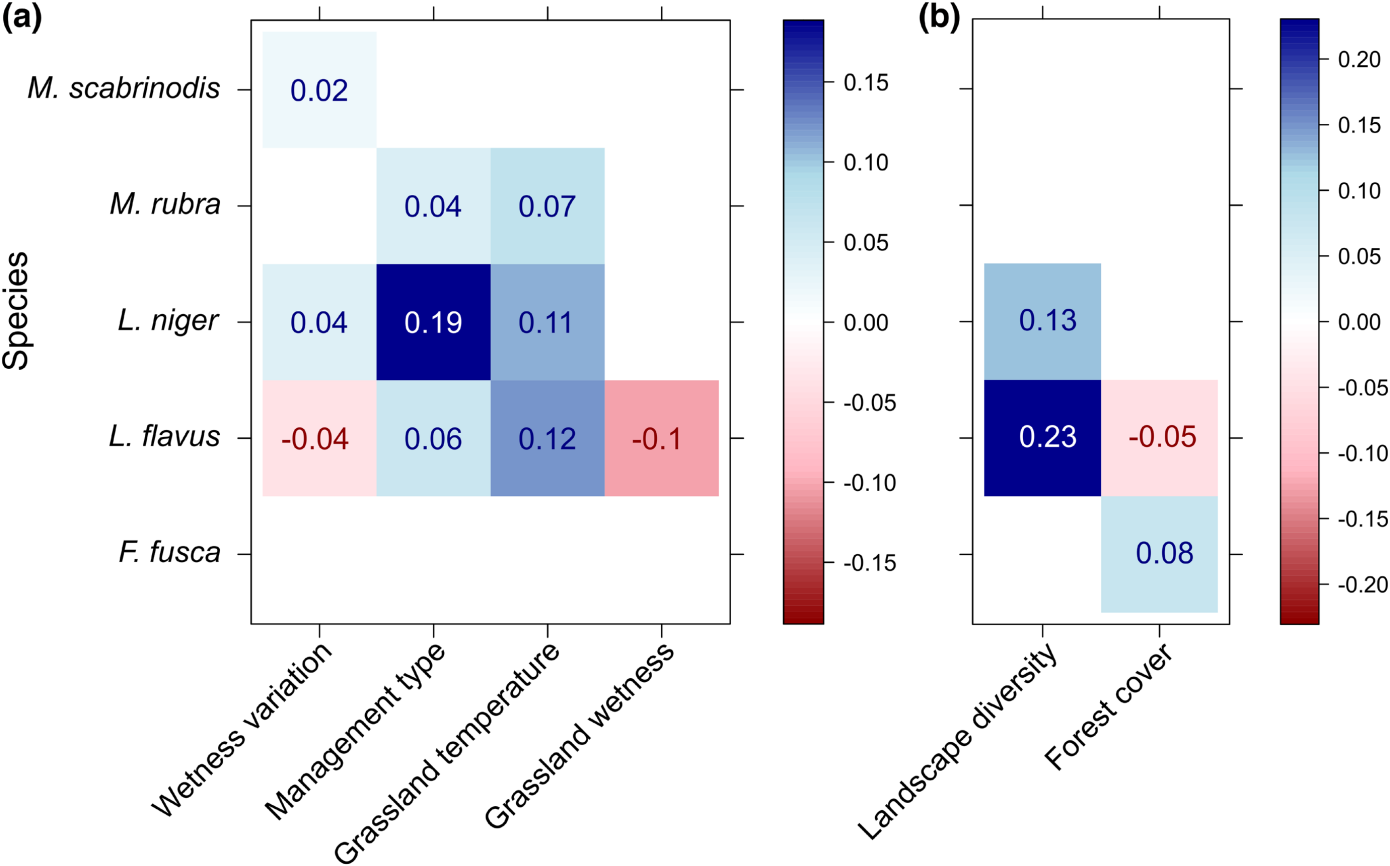
Responses of the most dense species to (a) local and (b) landscape environmental heterogeneity measures as standardized coefficients from multivariate SDMs (*R* ^2^ local = .23, *R* ^2^ landscape = .19). Size of coefficients can be interpreted as a measure of predictor importance. Color hue indicates positive (blue) or negative (red) species‐predictor association, while shading and numbers indicate the magnitude of association. Only significant predictors in Multivariate GLMs are shown.

Multivariate GLMs showed that the variation in community structure is explained by environmental heterogeneity at both local (LR = 191.9, *p* = .001) and landscape (LR = 64.1, *p* = .004) scale. Ant species composition was significantly affected by Wetness variation, Management type, Grassland temperature, Grassland wetness, Landscape diversity, and Forest cover predictors (*p* < .05; Table S3). Multivariate SDMs showed contrasting effects of Wetness variation on *Lasius* and *Myrmica* species, while Management type as pasture had an overall positive effect on species density, particularly strong for *L. niger* (Figure 2a). Warmer grassland temperatures had a positive effect on nest densities of *L. flavus*, *L. niger*, and *M. rubra*, while wetter grassland conditions had a negative effect on *L. flavus* nest density (Figure 2a). High Landscape diversity had a strong positive effect on nest densities of *Lasius* species, while Forest cover had contrasting effects on *L. flavus* and *F. fusca* densities (Figure 2b). Whole‐community nest density per grassland site increased with Wetness variation, Management type as pasture and Landscape diversity but decreased with the increase of Grassland wetness (Figure S2).

**FIGURE 3:**
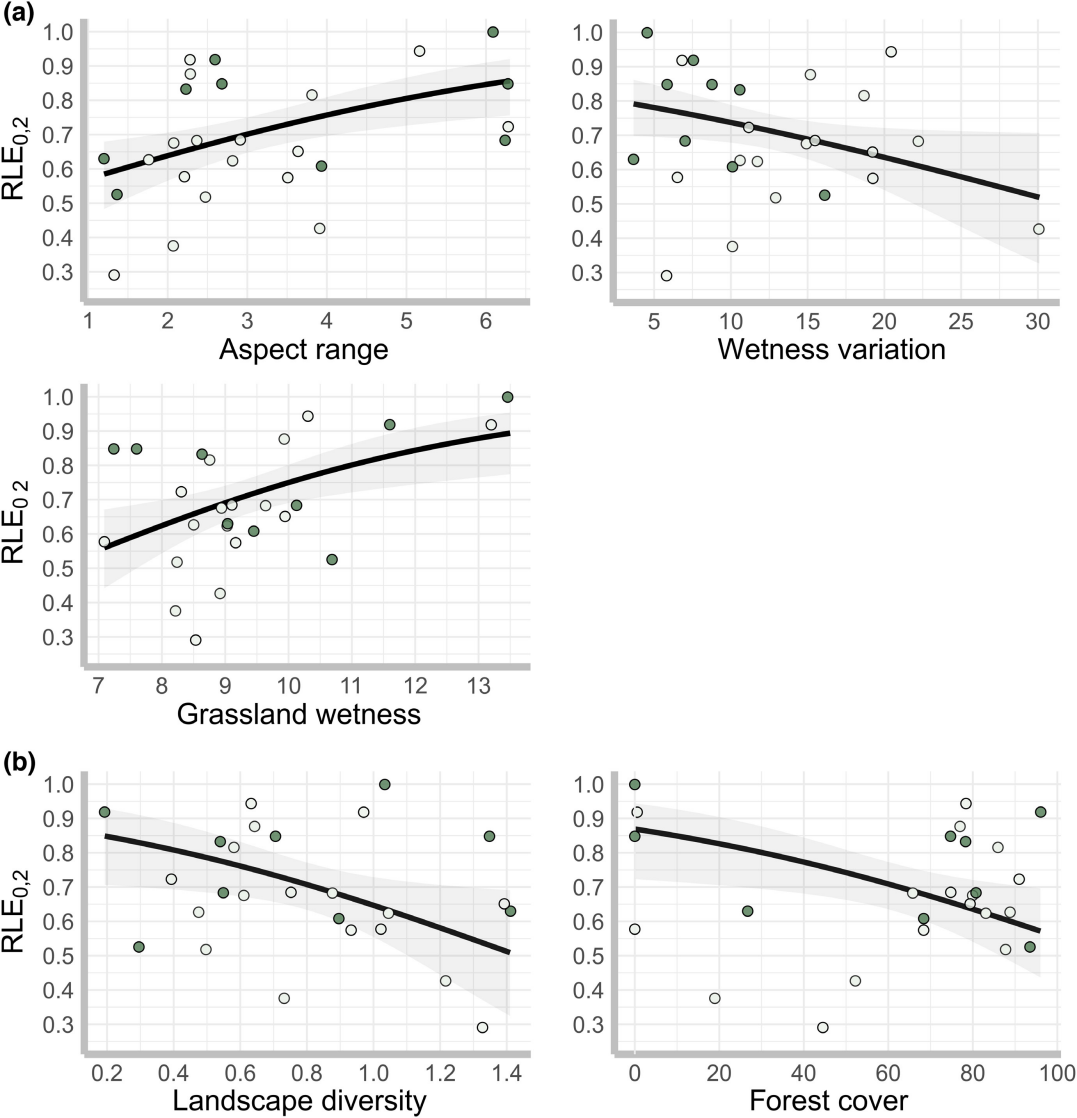
Effect of (a) local and (b) landscape environmental heterogeneity measures on ant community evenness. Solid line and shaded area show model estimated effect and confidence interval (95%) on RLE_0,2_ according to beta regressions (model detail in Table S4). Dots represent observations per grassland colorcoded by management type (Pasture: light‐filled; Meadow: dark‐filled). Only significant predictors in betaregression models are shown.

Beta regression models showed that community evenness was affected by local (logLR = 18.8, *p* = .001) and landscape (logLR = 11.29, *p* = .04) environmental heterogeneity measures. According with the likelihood ratio statistic (LR), the following predictors had significant effects on community evenness: Aspect range, Wetness variation, Grassland wetness, Landscape diversity, and Forest cover (*p* < .05; Table S4). At the local scale, RLE_0,2_ increased with the range of surface aspect (slope facing different directions) and Grassland wetness, but decreased with higher Wetness variation (Figure 3a). At the landscape scale, RLE_0,2_ decreased with the increase of Landscape diversity and Forest cover (Figure 3b).

**FIGURE 4:**
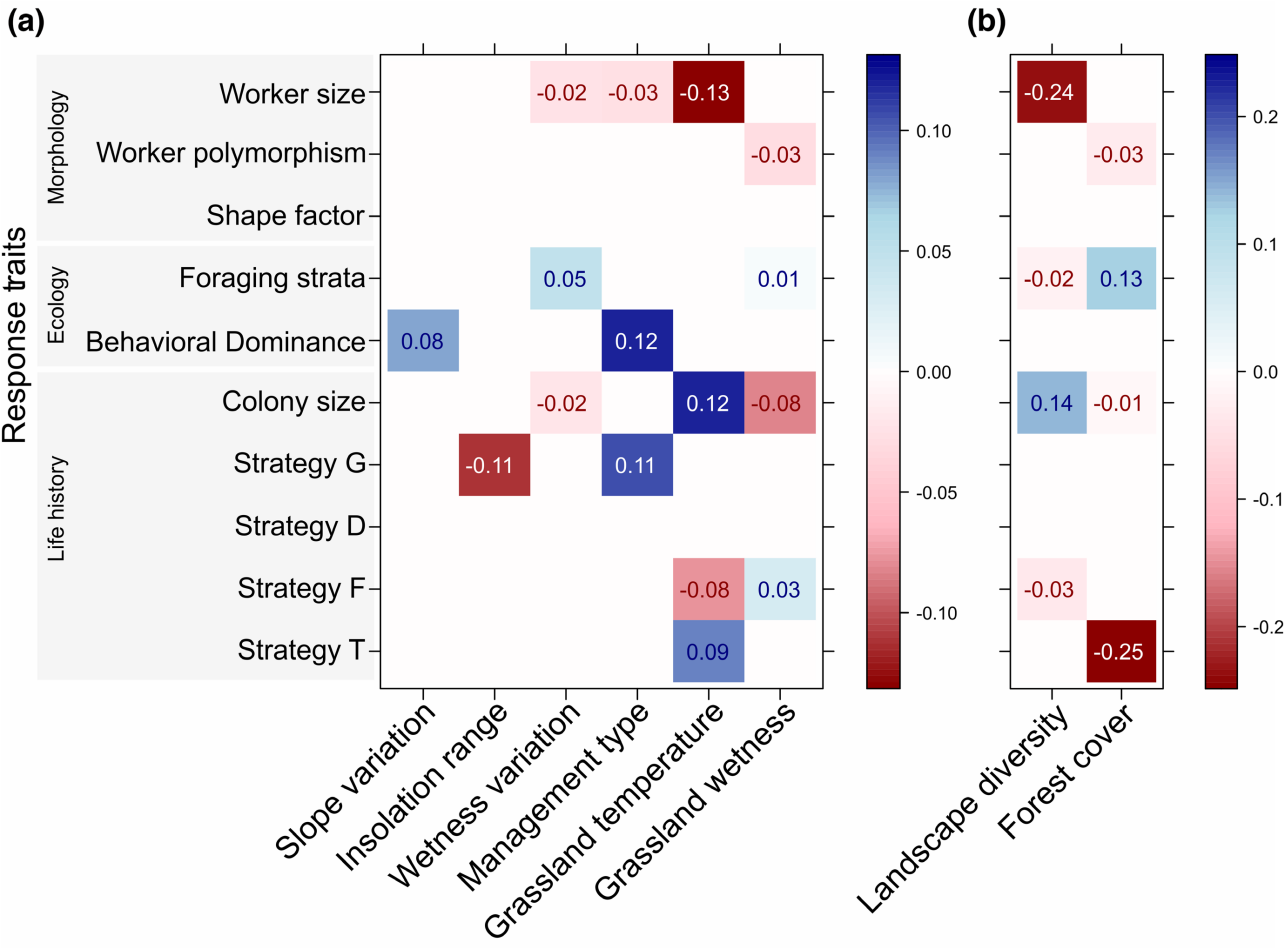
Interaction of species traits with (a) local and (b) landscape environmental heterogeneity measures. Standardized coefficients for the environment‐trait interaction terms in fourth‐corner models (GLM‐LASSO; *R* ^2^ local = .24, *R* ^2^ landscape = .21). Size of coefficients can be interpreted as a measure of importance of the interaction. Color hue indicates positive (blue) or negative (red) trait‐predictor association, while shading and numbers indicate the level of association.

Fourth‐corner models revealed a range of significant interactions between response traits and environmental predictors (Figure 4). The strongest community response to environmental heterogeneity measures was driven by worker size, colony size, behavioral dominance, and life history strategy traits (Figure 4). At the local scale, behavioral dominance was positively correlated with rugged slopes, while a generalist life history strategy was negatively correlated with insolation range (Figure 4a). Pastures managed by low‐intensity grazing promoted behaviorally dominant species with aggressive behavior and species with a generalist strategy for colony founding (Figure 4a). A wide range of traits were correlated with soil moisture measures but to a lesser extent (Figure 4a). Species with small workers and species limited by food during colony foundation decreased in warmer grassland sites (Figure 4a). Species with large colonies and species with time‐constrained and temperature limited nest foundation were favored by high grassland temperatures (Figure 4a). At the landscape level, the strongest environment‐trait interactions showed that higher landscape diversity had a negative effect on species with small workers, and higher forest cover in the surroundings hampered species with colony foundation dependent on temperature (Figure 4b).

## 4 DISCUSSION

We assessed the effects of local and landscape environmental heterogeneity on ant community structure in seminatural temperate grasslands. We found that extensively grazed pastures and within‐site heterogeneity in soil moisture and topographic exposition at the local scale, as well as diversity of land cover types and forest cover at the landscape scale affected ant community composition and evenness. Our findings additionally confirmed the importance of local mean temperature and soil moisture as environmental filters of ant species establishment in temperate grasslands (Dauber & Wolters, 2005; Heuss et al., 2019; Seifert, 2017). Furthermore, our trait‐based approach showed that the response of overall ant community structure to local and landscape environmental heterogeneity was mediated by a set of species traits including morphological, ecological, and life history features.

### 4.1 Effects of local and landscape heterogeneity on ant species composition

Ground‐dwelling ants are reportedly affected by habitat characteristics such as insolation and soil humidity as their soil‐nesting colonies depend on a certain envelope of temperature and soil humidity for foraging and successful rearing of the brood (Hölldobler & Wilson, 1990; Seifert, 2018; van Noordwijk et al., 2012). Changes in microhabitat conditions influence nesting sites supply for ants and consequently affect species composition by favoring the establishment and development of some species while culling or limiting others unable to tolerate such conditions (Czechowski et al., 2013; Dauber et al., 2005; Seifert, 2017). Such effects on species composition are also reflected on different levels of nest densities, where certain environmental conditions increase or decrease species‐specific nest densities (Seifert, 2017, 2018). In our study, within‐site variability of soil moisture (Wetness variation) affected ant species composition by increasing nest densities of several species and consequently whole‐community nest density per grassland site but also restricting densities of few others species (e.g., *L. flavus*), while an increase in Grassland wetness between sites explained the overall decrease of nest densities in our studied system. Although both results on soil moisture may seem contradictory, they should be interpreted as the effect of soil moisture as (i) an environmental heterogeneity measure reflecting variability within grasslands (Wetness variation) and (ii) an environmental means measure showing variability between grasslands sites (Grassland wetness; sensu Stark et al., 2017). High levels of soil moisture have been reported to adversely affect ant species richness and density in Central European grasslands, with most ant species having a narrow range of tolerance to water content in soils (Dahms et al., 2005; Dauber et al., 2005; Heuss et al., 2019; Seifert, 2017). In this sense, it seems likely that a higher environmental heterogeneity in terms of soil moisture (high Wetness variation) exerts a positive effect on ant communities within grassland sites by providing more niche‐spaces to be exploited by a wider group of species with different tolerances to wetness. On the contrary, the significant negative effect of Grassland wetness on ants likely reflects the gradient in humidity between grassland sites along the study area, where less humid grasslands (lower Grassland wetness) accommodated more diverse ant communities than more humid grassland sites (higher Grassland wetness). This is in line with a more regional‐scale pattern found across Central Europe, where ant diversity decreases from warm–dry to humid–cool grasslands (Dekoninck et al., 2007; Pérez‐Sánchez et al., 2018; Seifert, 2017). In contrast to soil moisture, the effect of Grassland temperature was more evident at species than at whole‐community level where nest densities of the reportedly thermophilic species *L. niger*, *L. flavus*, *M. rubra*, and *Formica cunicularia* (cf. Figure S3) increased along with Grassland temperature (Seifert, 2017, 2018).

In addition to the role of soil moisture, our findings showed that extensively managed pastures promote nest density of most of species compared with extensive meadows, which supports previous results highlighting the benefits of extensive grazing on biodiversity compared with annual mowing (Tälle et al., 2016). Although both extensive management types are expected to be beneficial for biodiversity in seminatural grasslands, the mechanisms behind their effect on grassland vegetation and fauna may differ (Lepš, 2014; Tälle et al., 2016). While low‐intensity (gradual but continuous) grazing by cattle creates a small‐scale mosaic of disturbances in soil and spatial structure of vegetation (vertical and horizontal), annual mowing homogenizes the vegetation structure through consistent and uniform biomass removal in a short period of time (Adler et al., 2001; Lepš, 2014; Olff & Ritchie, 1998; Tälle et al., 2016). Thus, grazing disturbance not only enables openness throughout the sward but also creates a variety of different microsites and microclimates suitable for a wider group of ground‐dwelling insects, including ants (Cole et al., 2010; Hoffmann, 2010; Jerrentrup et al., 2014). The differential effect of management types on grassland vegetation structure has been shown to affect ants indirectly by changing microhabitat and soil moisture conditions (Dahms et al., 2005; Dauber et al., 2005; Heuss et al., 2019; Pérez‐Sánchez et al., 2018), which correspond to our data showing higher within‐site soil moisture heterogeneity in pastures (Wetness variation: mean = 14.7, SD = 5.9) than in meadows (Wetness variation: mean = 8.9, SD = 4.5). Thus, both environmental heterogeneity measures, management type and soil moisture variability, may address the same synergistic effect of providing favorable microhabitat and soil conditions for ant communities.

At the landscape scale, a high diversity of land cover types had a general positive effect on species composition, while the influence of the surrounding forest varied among species. The effect of land cover heterogeneity on ant communities can be interpreted in two different ways. First, a higher variability in land cover types in the surroundings of the grassland sites may provide source habitats for new colonizing species, thereby enriching the local species pool (Benton et al., 2003; Öckinger et al., 2012). Second, a higher between‐habitat heterogeneity in the surroundings increases the range of abiotic conditions, particularly along the edges, thus causing ecotonal effects that may favor thermophilic species near open habitat edges (i.e., arable, built‐up land) or moist‐tolerant species near shaded edges (i.e., forest) within grassland communities (Dauber & Wolters, 2004; Frasconi Wendt et al., 2021). In our study region, the higher levels of landscape diversity are located at intermediate elevations where the land cover matrix transitions from a built‐up to forest‐dominated landscape and the proportions of neighboring arable lands and grasslands reach their peaks (Figure 1). Supporting the ecotonal effect, the observed species composition in many of these grasslands was characterized by species with different habitat preferences: *F. fusca* (woodland edges), *L. acervorum* (light forest), *F. cunicularia* (open verges), and *L. niger* (urban and disturbed open habitats; Seifert, 2018). The species‐specific effects of surrounding forests detected in our study further support this statement, as *F. fusca* density was positively affected by Forest cover while densities of *L. flavus* and *F. cunicularia* were negatively affected this land cover type (Figure S3).

### 4.2 Effects of local and landscape heterogeneity on ant community evenness

The effect of environmental heterogeneity on diversity has been explained by classical niche theory, where habitat heterogeneity is expected to increase species coexistence and therefore evenness (Stein & Kreft, 2015). However, it has been argued that the high diversity of species in seemingly homogeneous habitats cannot be explained exclusively by niche processes, but also by neutral or stochastic processes related to demography and dispersal events (Andersen, 2008; Brown et al., 2013; Hubbell, 2001). In this regard, evenness has been used as neutrality metric, meaning that habitats with high levels of community evenness are subject to more neutral than deterministic processes (Bar‐Massada et al., 2014; Schowalter, 2016). In our study system, 15 of 33 grassland sites accommodate even communities, suggesting that neutral processes may also contribute to structuring ant communities in addition to environmental heterogeneity. Neutrality has been suggested to be relevant for ant community assembly in discontinues areas at regional scale (Boet et al., 2020; Nooten & Guénard, 2022), and our study system, although at smaller spatial resolution, represents a landscape of discontinues grassland habitats within in a forest–urban matrix. The fact that ant response traits describing colony reproduction and dispersal constraints in seminatural grasslands (van Noordwijk et al., 2012) were important in our fourth‐corner analysis suggest that neutral processes, such as stochastic species dispersal, may contribute to the local assembly of ant communities. Nevertheless, these findings, although remarkably interesting, need to be considered with caution since neutrality levels in species‐poor communities are difficult to interpret as the lower bound of evenness decreases with richness (1/^0^ *D*), and in our studied system approximately 25% of the grassland sites showed communities with less than three ant species (Bar‐Massada et al., 2014; Jost, 2010).

In contrast to our expectations, the effect of environmental heterogeneity on ant community evenness was variable at local scale and negative at landscape scale. The effects of Wetness variation, Grassland wetness, and Landscape diversity on RLE_0,2_ were exactly opposite to those observed for species composition and density; hence, their interpretation is intimately related to our previous discussion. An increase in within‐site soil moisture variability and diversity of surrounding land cover types certainly supports whole‐community nest density, but also benefits numerical dominant species (i.e., *M. scabrinodis* or *L. niger*) that may lead to uneven or dominated ant communities in some grassland sites. Similarly, high levels of soil moisture may promote community evenness by limiting nest densities of numerical dominant ants in grasslands, which leads to a more even distribution of species densities within the community structure (c.f. sites in cluster 2, Figure S2.1). Model results additionally showed a positive effect of surface aspect range on community evenness. Our study system contains grasslands mostly facing from east to west through south azimuths. In grasslands facing east, the soil surface receives radiation earlier in the day when air temperature and evapotranspiration are lower leading to moister habitats (Ashcroft et al., 2008); while on west‐facing sites, the soil surface reaches maximum temperatures during the afternoon when the direct radiation is at its maximum creating warmer and drier habitats (Bennie et al., 2008). A higher range of surface exposure within grasslands sites creates highly heterogeneous habitats for ant species, thereby increasing species coexistence and promoting ant diversity and community evenness.

### 4.3 Community structure response to environmental heterogeneity through species traits

The community structure response to environmental conditions is the result of different sets of species (response) traits; therefore, quantifying how traits and environment interact provides a mechanistic understanding of community assembly (Lavorel & Garnier, 2002; Zirbel et al., 2017). Our results show that the response of ant community structure to local heterogeneity on topography, soil temperature, and management was strongly related to behavioral dominance and a generalist life history strategy. Behavioral dominance may be an advantageous trait for food acquisition and territory defense in open habitats characterized by patchy vegetation such as pastures with rugged slopes where the probability of intra‐ and interspecific competitive encounters is high (Savolainen & Vepsäläinen, 1988; Savolainen et al., 1989; Pérez‐Sánchez et al., 2018; but see Stuble et al., 2017). Similarly, species well adapted to deal with low food availability and variable soil temperatures during nest foundation (life history strategy G, according to van Noordwijk et al., 2012, cf. Table 2) may be favored in dynamic and heterogeneous habitats such as extensively managed pastures (Verberk et al., 2010). Surprisingly, we found a negative relationship between strategy G and within‐site insolation range, expected to be positive according to the environmental heterogeneity hypothesis. We attribute this result to the overpowering negative effect of insolation range (within‐site soil temperature variability) on *L. flavus* densities, rather than the cumulative effects on all species with life history strategy G (i.e., *L. niger* and *F. fusca*; van Noordwijk et al., 2012).

Community response to local grassland temperature and soil moisture was strongly related to worker and colony size traits and, to a lesser extent, to life history strategies F and T (cf. Table 2). Higher mean temperatures negatively affected worker size, but positively affected colony size. The relationship between body size and temperature has been traditionally related to the heat conservation hypothesis, that is, a larger body size has adaptive value in cold temperatures due to lower surface‐to‐volume ratios (Blackburn et al., 1999; Cushman et al., 1993). This hypothesis has been questioned for ants, as a larger body size in ectotherms also reduce the rate of heat gain, which is as important as decreasing heat loss (Cushman et al., 1993). However, it may be suitable at the colony level as long as (i) worker body size is positively correlated with colony size, and (ii) colony size is correlated with thermoregulatory capabilities (Cushman et al., 1993; Kaspari & Vargo, 1995). In our case, *F. fusca* meets such conditions and it highest nest densities were certainly registered at cooler temperatures in upland sites or grasslands surrounded by forest. Nevertheless, it is more likely that this particular result is influenced by species with small workers but large colonies such as *L. flavus* and *L. niger*, which showed high nest densities in grassland sites with high temperature and low wetness records. Although the ecological distribution of these three species frequently overlaps, their level of success in terms of nest numbers is related with different habitat conditions at local scale (Brian, 1964; Brian et al., 1965; Czechowski et al., 2013). In our studied sites, these species co‐occurred but rarely showed high nest density simultaneously, and communities with the highest density of either *L. flavus* or *L. niger* were grouped at opposite sides in the cluster analysis while communities having intermediate densities *F. fusca* were scattered along internal cluster groups (Box S2). Altogether, these results suggest a gradient on temperature and soil moisture conditions filtering ant species composition at local scale where the ecological mechanism behind the ant community response is related with the interplay of both worker size and colony size traits (Retana et al., 2015; Wiescher et al., 2012). Likewise, the interaction of life history strategies with temperature suggests that grassland sites with high mean air temperatures have a low nutrient content for ants (strategy F) but may shelter species whose nest foundation is highly dependent on soil temperature (strategy T; van Noordwijk et al., 2012).

The strongest interactions between traits and environment were detected at landscape scale, with a functional response to land cover types diversity similar to local grassland temperatures. Although both Grassland temperature and Landscape diversity predictors did not exceed the collinearity threshold (|*r*| < .7; Dormann et al., 2013), their effect on ant communities seems to be related. In a forest‐dominated landscape, a high number and even distribution of open land cover types (i.e., grasslands, arable lands, and urban areas) in the surroundings likely increases air and soil temperature of grasslands; while a decrease in grassland temperatures can be expected when surrounding landscape is dominated by forest due to shading (Figure S2; Krämer et al., 2012; Liivamägi et al., 2014; Öckinger et al., 2012). The strong and negative relationship between life history strategy T and Forest cover supports this statement, as species depending on high soil temperature for nest foundation showed low densities or were absent in grasslands surrounded completely or almost entirely by forest. In this sense, a high proportion of surrounding forests not only contributes to grassland isolation by imposing a physical barrier for species colonization but also limits abiotic conditions for the establishment of grassland specialist and thermophilic species (Krämer et al., 2012; Öckinger et al., 2012).

This study shows that the “filtering” effect of the abiotic environmental conditions is indeed sensitive to the spatial heterogeneity (Kraft et al., 2015). The impact of both abiotic conditions at local scale and biotic conditions at landscape scale on ant species composition was intimately related with spatial heterogeneity on habitat temperature and soil moisture. Our complementary trait‐based approach offered a deeper take of how environment heterogeneity drives ant community assembly at both scales, even revealed the filtering role of other environmental heterogeneity measures (e.g., Insolation range and Slope variation) not detected by the community composition approach. This shows that integrating both taxonomic and functional approaches is fundamental for ant community assessment at different scales (Carvalho et al., 2022; Frasconi Wendt et al., 2021; Heuss et al., 2019; Nooten & Guénard, 2022). A comprehensive interpretation of both diversity patterns and multiple trait environment interactions allows constructing a clearer picture of which and how ecological forces are operating behind the community assembly (Nooten & Guénard, 2022; Retana et al., 2015; Wiescher et al., 2012). Accordingly, our findings imply that a compromise between environmental heterogeneity at different scales and species response traits explains the ant community structure in temperate seminatural upland grasslands. This findings shed light on the site‐dependent response of ant diversity to grassland management found along European agricultural landscapes and suggest that, within a regional context, local site conditions regarding habitat temperature, soil moisture and surrounding landscape must be considered as key step prior to any assessment such relationship. Ultimately, a better knowledge about the ecological processes and the scales at which community drivers operate will contribute to establish more efficient agri‐environment schemes for the conservation of biodiversity in these agricultural landscapes (Costanza et al., 2011; Dauber et al., 2005; Öckinger et al., 2012).

## 5 CONCLUSIONS

Investigating the impacts of environmental heterogeneity on species diversity and functional traits at multiple spatial scales is crucial to better understand community patterns in ecology and is especially relevant for biodiversity conservation (Costanza et al., 2011; Ovaskainen et al., 2017). This study shows that local environmental heterogeneity within and between grasslands, and the surrounding landscape play a relevant deterministic role in structuring ant communities in temperate semi‐natural upland grasslands. Heterogeneity in habitat structure caused by grassland management (i.e., low‐intensity grazing) and within‐site heterogeneity in soil moisture were the most important local environmental measures shaping ant species composition and density. At the landscape scale, a high heterogeneity of land cover increased overall species density within grasslands, although the effect of each surrounding habitat type depended on species‐specific abiotic requirements. The variation of community evenness against environmental heterogeneity showed an opposite pattern compared with community composition due to the inherent relationship between richness and evenness (Jost, 2010). However, this approach revealed that a higher range of surface aspect (exposure of the slope) within grassland sites exerts a positive effect on community structure by promoting species coexistence. Overall, the proportion of even communities was high (54% of the surveyed sites) compared with uneven (35%) or dominated communities (11%), suggesting additional (neutral) processes operating on ant community assembly (Bar‐Massada et al., 2014). The response of ant community structure to environmental heterogeneity at both spatial scales was mediated by morphological (worker size), ecological (dominant behavior), and life history (colony size and strategies related with colony reproduction–dispersal‐foundation) traits. The interaction between the aforementioned response traits and the environment framed in a fourth‐corner scheme proved to be a valuable approach for understanding how community structure is driven by environmental heterogeneity. We conclude that ant communities in seminatural grasslands are not only driven by environmental heterogeneity but also by complementary neutral processes related to stochastic habitat colonization, as dispersal events between isolated grasslands are limited in a forest‐dominated landscape. Both environmental heterogeneity and stochastic explanations are not exclusive and may represent opposite ends of the same continuous gradient denoting the relative contributions of both niche and neutral processes to the assembly of ant communities in temperate seminatural grasslands (sensu Gravel et al., 2006).

## AUTHOR CONTRIBUTIONS

**Antonio J. Pérez‐Sánchez:** Conceptualization (lead); data curation (lead); formal analysis (lead); funding acquisition (equal); methodology (equal); writing – original draft (lead). **Anett Schibalski:** Data curation (supporting); formal analysis (equal); writing – review and editing (supporting). **Boris Schröder:** Conceptualization (supporting); formal analysis (supporting); resources (supporting); supervision (supporting); writing – review and editing (equal). **Sebastian Klimek:** Conceptualization (supporting); methodology (equal); resources (supporting); supervision (equal); writing – review and editing (equal). **Jens Dauber:** Conceptualization (supporting); funding acquisition (equal); methodology (equal); resources (equal); supervision (lead); writing – review and editing (equal).

## FUNDING INFORMATION

Open Access funding enabled and organized by Projekt DEAL.

## Supporting information


Appendix S1

## Data Availability

The data that support the findings of this study will be openly available in Dryad https://doi.org/10.5061/dryad.5dv41ns8c.
