# Corrigendum

**DOI:** 10.1002/ece3.9983

**Published:** 2023-04-07

In the recent article by Pérez‐Sánchez et al. (2023), the authors would like to add the ORCID IDs for the following authors:

Anett Schibalski: https://orcid.org/0000‐0002‐1686‐8811

Boris Schröder: https://orcid.org/0000‐0002‐8577‐7980

Sebastian Klimek: https://orcid.org/0000‐0002‐2544‐640X

Jens Dauber: https://orcid.org/0000‐0002‐3420‐0380
