# Supplementary material for: Local and landscape environmental heterogeneity drive ant community structure in temperate seminatural upland grasslands
Source: Ecol Evol. 2023 Mar 19;13(3):e9889. doi: 10.1002/ece3.9889 (PMC10025078; doi:10.1002/ece3.9889)
Supplement: Supplementary file 1 — Appendix S1 [file ECE3-13-e9889-s001.pdf]

Local and landscape environmental heterogeneity drive ant community structure in temperate semi-natural upland grasslands

Antonio J. Pérez-Sánchez[1,2], Anett Schibalski[3], Boris Schröder[3,4], Sebastian Klimek[1], Jens Dauber[1,2]

[1] Thünen Institute of Biodiversity, Braunschweig, Germany.
[2] Biodiversity of Agricultural Landscapes, Institute of Geoecology, Technische Universität Braunschweig, Braunschweig, Germany.
[3] Landscape Ecology and Environmental Systems Analysis, Institute of Geoecology, Technische Universität Braunschweig, Braunschweig, Germany.
[4] Berlin-Brandenburg Institute of Advance Biodiversity Research (BBIB), Berlin, Germany.

AppendixS1

Table S1. Number of Seifert-plots per grassland site.

Grassland ID	Number of Seifert-plots	Grassland area (ha)
G1	3	3.01
G3	3	3.03
G5	2	3.18
G6	1	0.77
G7	2	1.40
G10	2	3.67
G12	2	1.98
G14	3	3.81
G20	1	1.90
G21	2	3.38
G22	2	1.70
G24	2	1.90
G46	3	6.17
G48	1	1.18
G49	1	0.75
G50	2	1.25
G52	2	1.42
G55	1	0.92
G56	1	1.50
G57	1	1.09
G63	2	0.83
G86	2	3.10
G91	1	0.51
G110	3	2.75
G115	2	1.13
G128	1	0.21
G156	2	0.94
G200	3	5.95
G201	1	1.39
G300	2	2.02
G443	2	5.20
G538	2	6.31
G639	1	1.88

Note: Covering the entire grassland extension for sampling was not always possible due to the presence of cattle in subsections of pastures or restricted access caused by rugged topography and flooded areas. The number of Seifert-plots established for ant survey depended on these spatial limitations and grassland size. One Seifert-plot was performed in smaller grasslands sites (< 0.77 ha), one-two plots intermediate size grasslands (0.8-1.9 ha), and two-three plots in larger sites (> 1.9 ha). On average, our ant survey procedure covered 20% of grassland sites area.

Box S1. Integrated nest density calculation within grassland sites.

Nest density calculation. Nest abundance from each Seifert-plot component (*S*-, *Q*-, and *SI*-) was combined into a final integrated species-specific density (ISSD) which represents the nest density of a species within 100 m^2 (Seifert 2017). The ISSD per species is calculated as the sum of nests found in the *S*-, *Q*- and *SI*- sampling areas divided by the *pseudo-area* of the "recording group" (*RG*) to which a particular species belongs (Eq. 1; Seifert 2017). The *RG* is a generalization of how perceptible a nest is accordance with the ant species biology. The assignment of a species into a given *RG* describes the probability of finding a nest in each sampling level (*S*-, *Q*-, and *SI*-), and such probability is determined by nests position, type, size and density (Seifert 2017). Based on almost four decades of research on Central European ants, Seifert (2017) defined five *RG*s (*i*-*v*) ranking from lowest to largest perceptibility. The *pseudo-area* is calculated for each *RG* separately and provides a measure of the total intensity of investigation on a Seifert-plot per *RG*. This parameter may be understood as the area equivalent needed in a specific *RG* to find the sum of nests recorded by *S*-, *Q*- and *SI*-search (Seifert 2017). The *pseudo-area* is defined as the sum of all nests detected in all search levels divided by a fixed value per RG, the recording-group-specific total density or FRSD (Eq. 2; Seifert 2017). The FRSD is the number of nests of a specific *RG* expected to occur in 100 m^2, based on the total number of nest of all species occurring in such RG and weighted by the sampling area (Eq. 3). The sampling area employed in Eq. 3 is subedited to the *RG* (See Seifert 2017 for more details).

$$Eq.1\; ISSD = \frac{S+Q+SI}{pseudo\text{-}area_{RG}} \quad Eq.2\; pseudo\text{-}area = \frac{S+Q+SI}{FRSD} \quad Eq.3\; FRSD = \frac{nestsx100m^2}{Samplingaream^2}$$

As a short example let us say that we are interested in calculating nest densities of *M. rubra* per grassland sites. Our estimations would be focused on the RG *iii* (common grassland species; Seifert 2017), and FRSD and *pseudo-area* would be based on *S*- sampling area (64 m^2). Thus, in an hypothetical community of five species and 11 nests resulting from a Seifert-plot with *S*= 3 nests (2 *M. rubra*), *Q*= 4 nests (1 *M. rubra*) and *SI*= 4 nests (0 *M. rubra*), the FRSD and *pseudo-area* will be 4.688 nests/100 m^2 and 2.347 m^2 respectively, and the integrated species-specific density (ISSD) for *M. rubra* will be 1.278 nests/100 m^2. Finally, following the Seifert (2017) method, we used accumulation and extrapolation curves for sampling completeness assessment. This method aims to estimate the species number S found in a certain habitat as a function of sampling effort E (quantified by m^2), and used a natural logarithmic function (S = a Ln E + b) for constructing extrapolation curves. For a more details consult Seifert 2017.

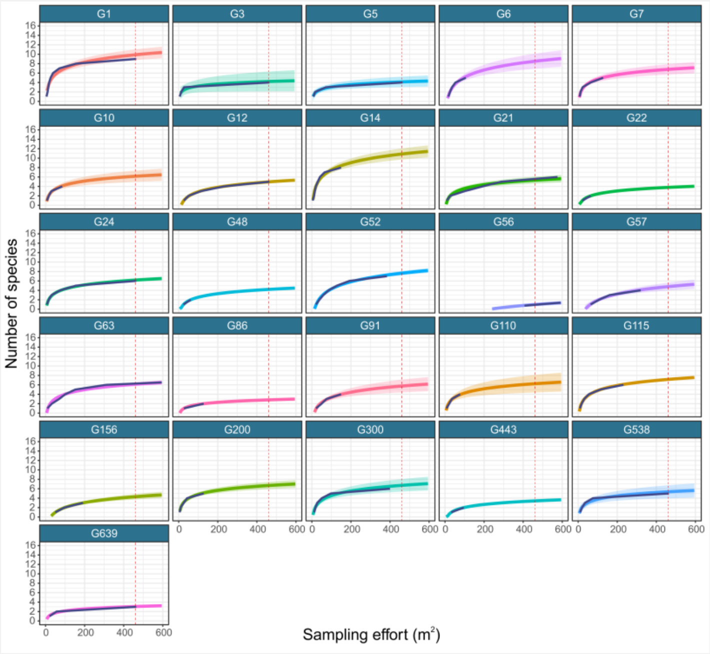

The figure shows accumulation (blue line) and extrapolation curves (colored line) with 95% confidence interval (shaded area) for each grassland site. Sites with only one recorded species were excluded (G46, G48, G49 G50, and G201). Between 60% and 98% of the ant fauna were recorded per grassland site based on logarithmic extrapolation and minimum sampling effort E employed (460 m^2; red dotted line).

References

Seifert B (2017). The ecology of Central European non-arboreal ants – 37 years of a broad-spectrum analysis under permanent taxonomic control. Soil Organisms 89(1): 1-67.

Table S2. Description of the response traits used in the Fourth-corner analysis.

**Trait**		**Description**	**Data type**	**Data range**
**Morphology**				
Worker size		Worker body size from the tip of mandibles to tip of the gaster (mm). Mean values from 10 individuals per colony complemented with measurements available in Cushman et al. (1993) and Seifert (2018).	Continuous	2.9 to 7.5
Worker polymorphism		Range of worker size divided by the mean of worker size. Relates to the breath of functional roles performed by colony. Source: Arnan et al. (2017).	Continuous	0.1 to 1
Shape factor		A body shape measurement based on compactness. Allows grouping species with similar basic morphology. Larger values indicate a more thick-set or compact body shape. Source: Seifert (2017)	Continuous	1.3 to 2.6
**Ecology**				
Foraging strata		Calculated as the sum of the probability of worker to forage in different vertical strata, where the value for each stratum is multiplied by a specific factor. Positive values indicate higher probability foraging above the ground; negative values indicate foraging under the surface. Source: Seifert (2017), Heuss et al. (2019).	Continuous	-3 to 1.3
Dominant behavior		Presence/absence of behavioral dominance. This trait indicates the species ability to gain access to food resources. Refers to the influence of one species on a different one when acquiring food resources and thus the ability to gain access to food resources. Source: Savolaineb and Vepsäläinen (1988), Savolainen et al. (1989), Retana et al. (2015)	Binary	0, 1
**Life history**				
Colony size		Mean number of worker per colony (ln-transformed). Source: Arnan et al. (2017) and Seifert (2018).	Continuous	2.3 to 11
Life history strategies *	G	Consists of species with claustral founding mode with a broader time-window for initial larval development. Comprises more generalist species that are reasonably good dispersers and well adapted to cope with both low food availability and low temperature.	Categorical	N = 3
	D	Species with nest-splitting or parasitic founding as main nest founding mode, which are poor dispersers between habitats. Due to the availability of workers during the nest founding phase they are much less affected by low food availabilities and low soil temperatures during nest founding.		N = 3
	F	Species with semi-claustral founding mode that are reasonably good dispersers but are limited to sites with high food availability as their queens have to forage by themselves to feed their first batch of workers.		N = 8
	T	Consists on claustral species with a very narrow time-window for initial larval development. Since claustral queens feed their first worker batch from their own nutritional reserves, they are not affected by the food availability during nest founding but by the time available for development of the first batch. Therefore, these time-constrained species are restricted to warm site with high soil temperatures during nest founding.		N = 2

* Source: van Noordwijk et al. 2012

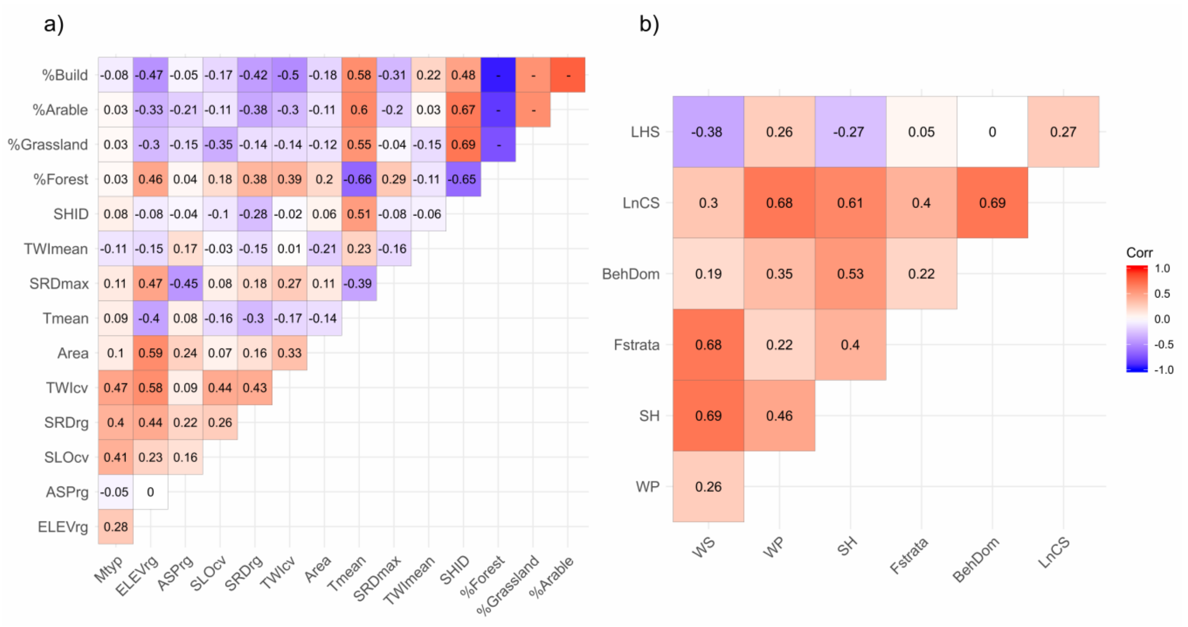

Figure S1. Correlation matrix of a) environmental variables and b) response traits based on Pearson correlation coefficient (*r*).

Variable coding: (a) Mtyp, management type (transformed to numeric variable for correlation coefficient calculations); ELEVrg, elevation range; ASPrg, surface aspect; SLOcv, coefficient of variation of slope; SRDrg, insolation range; TWIcv, coefficient of variation of the topographic wetness index; SHID, Shannon index of diversity of land cover types; %Forest, %Grassland, %Built-up, %Arable, percentage of land cover types. (b) WS, worker size; WP, worker polymorphism; SH, shape factor; Fstrata, foraging strata; BehDom, dominant behavior; LnCS, Colony size; and LHS, life history strategies (transformed to numeric variable for correlation coefficient calculations).

Box S2. Community structure descriptive results

Cluster analysis revealed a pattern of species composition within grassland sites related to management type, total nest density, and their most dense species or group of species (Figure A2.1). The first division (*k*=2) generated one group of communities with high nest density in sites predominately managed as pasture and another group of communities with low density located in sites either managed as pasture or meadow (Figure A2.1). In the first group, a second division (*k*=3) separated uneven communities with high *M. scabrinodis* density (cluster 1) from sites with intermediate densities of this species but relatively high densities of other species (clusters 3, 6; Figure A2.1). A third division (*k*=4) in the second group separated grassland sites with high nest densities of *L. flavus* (cluster 4) from sites with even communities of rather low nest density (cluster 2; Figure A2.1), with a further division (*k*=5) of this branch grouping sites where *M. rubra* was the most dense species (cluster 5). A fifth division (*k*=6) split grasslands sites with intermediate densities of *M. scabrinodis* (cluster 3) from sites with high densities of *L. niger* (cluster 6). Internal cluster validation showed a decrease of the level of goodness and misplaced grassland sites with the increase of *k* clusters generation (Table A2.1). We considered *k*=6 as the most appropriated number of clusters with an overall S_i= 0.38 and non-misplaced data points. Divisions with $k > 7$ led to $S_i = 0$ within clusters suggesting that the algorithm does not succeed in finding any 'natural' clustering (Rousseeuw 1987).

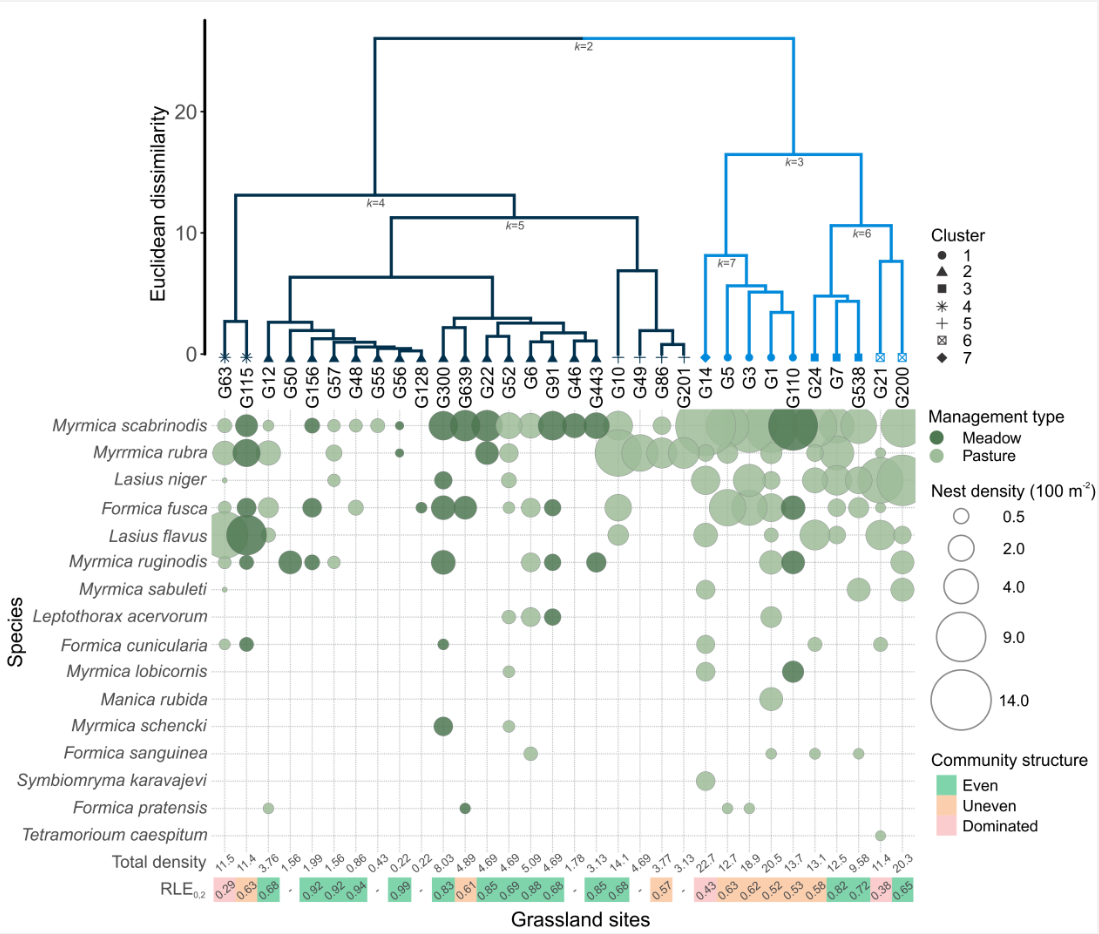

Figure S2.1. Dendogram represents a hierarchical cluster analysis based on species density in 32 grasslands sites. Color-coded clusters highlight the first group division, k values illustrate the number of cluster groups generated per division (step-wise), symbols at the cluster's end show the final clustering-group in the analysis, and codes (leaf labels) at dendrogram's bottom show the grassland site ID (see Figure 1). The bubble chart represents the species density matrix, arranged from high to low nest density per species. Total species density and $RLE_{0.2}$ values are also provided for each grassland site. Evenness analysis was not performed in grassland sites with only one species.

Table S2.1. Internal cluster validation based on Silhouette (*Si*) analysis. Table presents the average Si coefficient (Overall *Si*), number of mis-clustered data points (sites) and data point identity per each cluster generation (*k*).

Number of clusters (*k*)	Overall S_i	mis-clustered sites	Grassland site code
k=2	0.43	3	G21, G538, G7
k=3	0.40	3	G24, G7, G538
k=4	0.40	3	G24, G7, G538
k=5	0.39	3	G24, G7, G538
k=6	0.38	1*	G5
k=7	0.37	0	-

* We considered G5 observation as appropriately clustered following a parsimonious approach as its silhouette coefficient for was rather low (*Si* = -0.09).

Note: The average silhouette criterion calculates the overall silhouette coefficient (*Si*) per division (*k*- cluster generation) to assess the appropriate number of clusters (Rousseeuw 1987, Kaoungku et al. 2018). *Si* values ranges from -1 (data points placed in the wrong cluster) to 1 (data points well clustered), where small values (around 0) means that the observation lies between two clusters.

References

Kaoungku, N. et al. (2018) ‘The Silhouette Width Criterion for Clustering and Association Mining to Select Image Features’, International Journal of Machine Learning and Computing, 8, pp. 69–73.

Rousseeuw, P. J. (1987) ‘Silhouettes: A graphical aid to the interpretation and validation of cluster analysis’, Journal of Computational and Applied Mathematics, 20(C), pp. 53–65. doi: 10.1016/0377-0427(87)90125-7.

Table S3. Ant community composition variation explained by local and landscape environmental heterogeneity measures. Multivariate GLMs for species density were tested by means of deviance analysis (likelihood- ratio test, Monte Carlo bootstrapping, 999 resamples). Statistically significant results ($P < 0.05$) are shown in bold.

	**LR**	**Res. df**	**Deviance**	**P**
**Local environmental heterogeneity** (R^2= 0.20, AIC= 992.9)				
Slope variation	47.2	29	20.0	0.13
Insolation range	46.6	28	15.9	0.47
Wetness variation	73.5	27	73.5	**0.001**
Management type	39.3	30	25.6	**0.03**
Grassland temperature	53.4	26	33.8	**0.002**
Grassland wetness	46	25	23.1	**0.04**
**Landscape environmental heterogeneity** (R^2= 0.02, AIC= 992.7)				
Landscape diversity	34.9	30	29.7	**0.01**
Forest cover	34.4	29	34.4	**0.003**

Table S4. Ant community evenness variation explained by local and landscape environmental heterogeneity measures. Beta regression models for relative evenness ($RLE_{0,2}$) were compared using likelihood ratio tests (Chi-squared distribution). Statistically significant results ($P < 0.05$) are shown in bold.

	**logLR**	**Res. df**	χ^2	**P**
**Local environmental heterogeneity** (R^2= 0.65 , AIC= -29.4)				
Aspect range	17.7	7	17.9	**0.006**
Insolation range	20.1	6	3.13	0.08
Wetness variation	19.6	5	4.14	**0.04**
Area	20.2	4	2.89	0.09
Grassland wetness	17.0	3	9.37	**0.002**
**Landscape environmental heterogeneity** (R^2= 0.17, AIC= -14.6)				
Landscape diversity	8.3	4	6	**0.01**
Forest cover	9.17	3	4.23	**0.04**

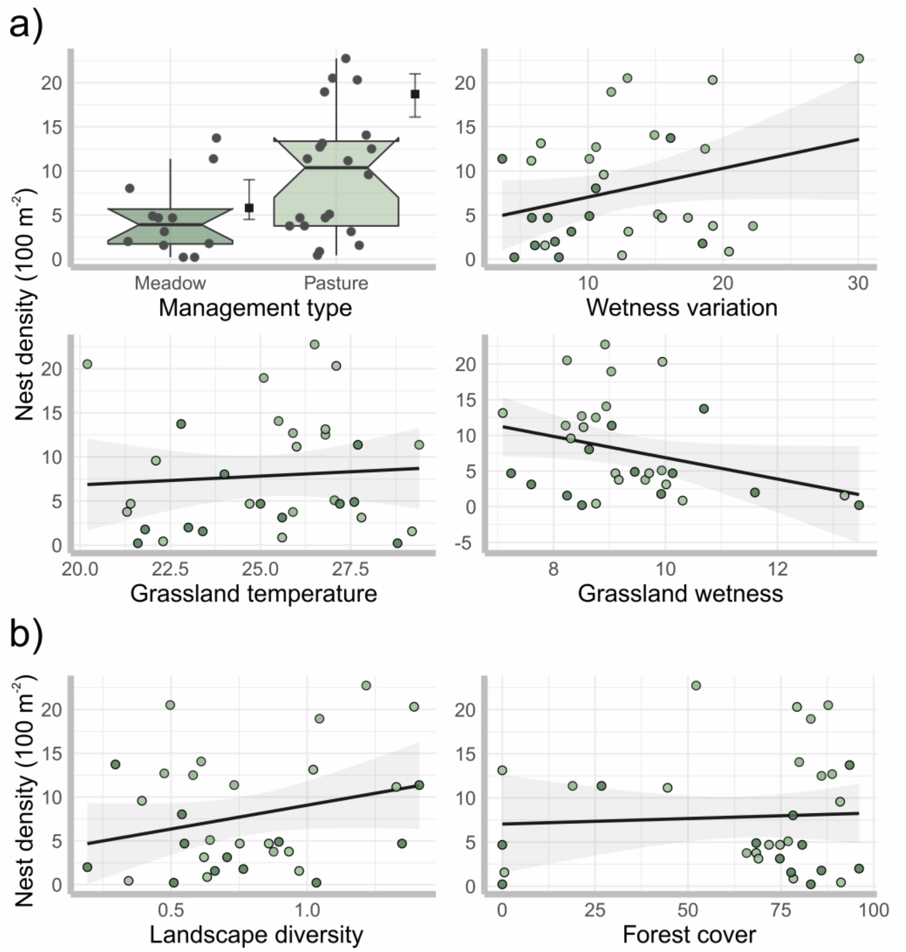

Figure S2. Relationship between whole-community nest density and significant a) local and b) landscape environmental heterogeneity measures. Line and shaded area (square and error bar in the boxplot) show estimated effect and confidence interval (95%) according GLMs, dots represent observations per grassland color-coded by management type (pasture: light-filled; meadow: dark-filled).

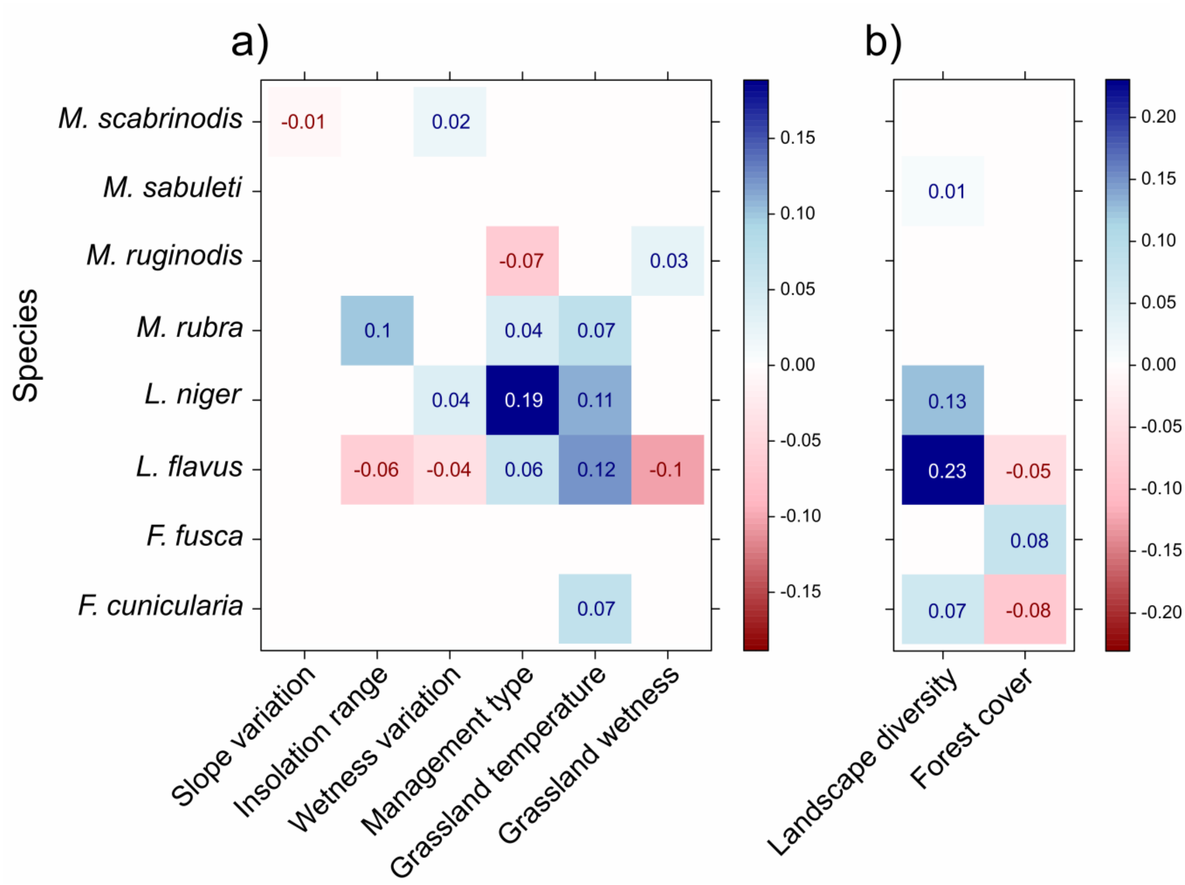

Figure S3. Ant species responses to a) local and b) landscape environmental heterogeneity measures as standardized coefficients from multivariate SDMs ($R^2_{local} = 0.23$, $R^2_{landscape} = 0.19$). Size of coefficients can be interpreted as a measure of predictor importance. Color hue indicates positive (blue) or negative (red) species-predictor association, while shading indicates the magnitude of association. All predictors fitted in SDMs are shown.
